# COVID-19 and hospital management costs: the Italian experience

**DOI:** 10.1186/s12913-022-08365-9

**Published:** 2022-08-04

**Authors:** Emanuela Foglia, Lucrezia Ferrario, Fabrizio Schettini, M. Beatrice Pagani, Martina Dalla Bona, Emanuele Porazzi

**Affiliations:** Healthcare Datascience Lab (HD LAB), Centre for Health Economics, Social and Health Care Management, Carlo Cattaneo – LIUC University, 21053 Castellanza, Italy

**Keywords:** COVID-19, Economic evaluation, Time-driven activity-based costing analysis, Italy, Hospitalization costs

## Abstract

**Background:**

This article investigates the hospital costs related to the management of COVID-19 positive patients, requiring a hospitalization (from the positivity confirmation to discharge, including rehabilitation activities).

**Methods:**

A time-driven activity-based costing analysis, grounding on administrative and accounting flows provided by the management control, was implemented to define costs related to the hospital management of COVID-19 positive patients, according to real-word data, derived from six public Italian Hospitals, in 2020.

**Results:**

Results reported that the higher the complexity of care, the higher the hospitalization cost per day (low-complexity = €475.86; medium-complexity = €700.20; high-complexity = €1,401.65). Focusing on the entire clinical pathway, the overall resources absorption, with the inclusion of rehabilitation costs, ranged from 6,198.02€ to 32,141.20€, dependent from the patient’s clinical condition.

**Conclusions:**

Data could represent the baseline cost for COVID-19 hospital management, thus being useful for the further development of proper reimbursement tariffs devoted to hospitalized infected patients.

## Background

Coronavirus disease-2019 (COVID-19) is a novel major healthcare problem caused by severe acute respiratory syndrome coronavirus-2 (SARS-CoV-2), that rapidly spread around the globe. The dramatic increase in the number of cases and deaths have placed tremendous strain on healthcare systems worldwide. In the Italian setting, the confirmation of the first case of COVID-19 in Codogno Hospital, in February 2020, represents the starting point for extraordinary measures of national and regional management, evolving according to the infections registered daily.

Healthcare organizations have thus been required to revisit their internal models and processes, for the improvement of services and procedures efficiency, and for the management of the tension between safety needs and the unexpected priority to redesign and reengineer the delivery of care processes [1, 2]. The Hospital Crisis Boards have entirely remodeled their healthcare delivery services, providing a more efficient discharge processing of patients, freeing-up as many beds as possible in the wards, especially those characterized by medium or high complexity of care, devoted to patients with acute and severe respiratory syndromes [3]. Within the Italian epidemic peaks, the virus overloaded hospitals, with the saturation of hospital beds, the increasingly demand for medical devices and equipment (from complex equipment like ventilators or extracorporeal membrane oxygenation devices, to hospital staples, like saline drip bags), as well as the lack of trained healthcare professionals.

In this view, the management of both clinical and organizational issues related to COVID-19 has led all levels, operating in a context of radical uncertainty, highlighting the importance of being able to make decisions relying on valid, shared, provided in real time, understandable data, emphasizing the relevance of real-world information and, at the same time, the urgent priority to handle and manage this information seriously [4–6].

In this complex scenario, literature on the topic focused on efficacy and cost-effectiveness nature of the therapeutic strategy potentially used for the treatment and cure of COVID-19 positive patients [7–12], or on the presentation of forecasting models, estimating healthcare costs based on assumptions and input data derived from scientific evidence [13]. Another hot topic of discussion was the impact analysis of the different public healthcare strategies developed for fighting the virus, based on dynamic models stressing the epidemiological nature of the infection and comparing the outcomes of different containment policies, without paying attention to healthcare hospitals’ expenditures and budgets [14–17]. Indeed, as the real problem was saving lives and mitigating the spread of the virus, little attention was initially paid to hospital accountability and economic reporting aspects, grounding on country-oriented and real-life information for the economic evaluation of the entire clinical pathway of COVID-19 positive patients.

According to the above, today the investigation of the economic resources absorption is becoming an urgent priority, concerning the management of COVID-19 positive patients requiring a hospitalization, to support the decision-making process, and to implement adequate healthcare planning policies. In this regard, the estimation of costs could represent a relevant element not only for an adequate healthcare resources’ allocation, but also for adopting measures against major healthcare events and pandemic situations. Furthermore, the coverage of the above knowledge economic gap represents a significant issue to be explored, to support the production of adequate reimbursement tariffs devoted to COVID-19 positive hospitalized patients, due to the current lack of a specific ICD (International Classification of Diseases) code to produce the proper Diagnosis Related Group (DRG), overcoming the current approach of tariffs assimilation, not always covering all the economic efforts sustained by hospitals.

Moving on from these premises, the study aims at determining the hospital costs devoted to the management of COVID-19 positive patients, considering both a single hospitalization day and the entire hospital stay, for the identification of the overall resources’ absorption, based on the COVID-19 positive patients’ severity and clinical conditions, thus also considering the rehabilitative pathway, after the hospital discharge. The achievement of this objective would be useful to try answering to the following challenging research questions: *i)* How many resources does the hospital management of a COVID-19 positive patient absorb, within the different care intensity settings (low, medium or high-care intensity), considering a single hospitalization day? *ii)* How many resources does the hospital management of a COVID-19 positive patient absorb, considering the entire hospital stay, from the confirmation of COVID-19 positivity to discharge, with also the inclusion of the rehabilitation pathway?

## Methods

A process mapping technique, grounding on a time-driven activity-based costing approach – TDABC—was implemented for the definition of the economic resources’ absorption devoted to COVID-19 positive patients [18, 19]. With the application of this microanalytic approach, it was possible to measure the cost of all the resources used to treat the COVID-19 patient medical conditions [20, 21], thus defining the care delivery value chain, with all the activities performed over the entire care cycle [22, 23].

In particular, the assessment considered the entire hospital stay, from the confirmation of COVID-19 positivity to the discharge, with the inclusion of the rehabilitative activities, according to real-world data collected in 6 public Italian Hospitals managing COVID patients, from February to December 2020. Coherently to the fact that in the considered time-horizon 87% of the confirmed COVID-19 cases were inhabitants of northern regions (accounting for the 55% of the Italian population) [24], the present study involved hospitals referring to Lombardy, Piedmont, and Veneto Regions.

The analysis considered the hospital point of view, in terms of identification of the economic resources’ absorption directly sustained by hospitals, in taking in charge infected patients. Process maps were developed based on the severity of the infection requiring a different hospitalization and were used to identify all resources consumed during the care pathway.

The clinical pathway of COVID-19 positive patients was economically evaluated, considering the following healthcare items of expenditure, representing the input data of the economic evaluation conducted.

i) *Human resources*, in terms of assistance minutes spent by clinicians or nurses, at the bed of the patients, valorized in accordance with the Italian National Labor Contracts per professional class, considered as labor costs. This approach was useful to allocate a cost for each activity in terms of the work factor, thus calculating the cost per minute for each healthcare professional involved in the treatment and care of the hospitalized COVID-19 patients. Labor costs was then correlated to the overall hospital length of stay, considering the entire number of days spent by the COVID-19 positive patients, from the confirmation of COVID-19 positivity to the day of discharge, also including the rehabilitative activities duration.
ii) *Laboratory exams and diagnostic procedures* conducted during the hospitalization, in terms of quantity and typology of procedures derived from the hospitals’ flows and divided per typology of procedure. Each one was multiplied for the related standard cost derived from the national reimbursement outpatients tariff, valid also for the years 2022, to define the total costs.
iii) *Drugs administered to the patients*, in terms of dosage and duration. For the definition of the total drug cost, the cost of each medication was multiplied by the related hospital acquisition cost, considering the duration of the drugs’ administration cycle during the length of stay.
iv) *Oxygen therapy*, analyzing both invasive and non-invasive ventilation procedures, thus evaluating the overall therapy duration, as well as the medical technologies utilized, according to the related time of use.
v) *Equipment*, in terms of typology of equipment implemented for the management of COVID-19 positive patients. For the economic evaluation of the equipment, the analysis did not consider the equipment already present within the investigated settings, as they are already amply depreciated. For the new equipment (i.e. equipment directly purchased for the pandemic situation), a life-cycle equal to 5 years was considered, and a VAT rate equal to 22% was integrated, thus also assessing the considered equipment use cost per minute, for the treatment and care of COVID-19 positive patients.
vi) *Personal Protective Equipment (PPE)*, considering the number of the different PPE consumed by both the healthcare professionals and the patients.
vii) *Cleaning services and meals*, attributable to the single COVID-19 positive patients, regarding the related length of stay.
viii) *General and fixed costs*, consisting of all those costs different from labor factors, consumables, and equipment usage, being necessary to taking in charge patients because they provide the logistic and infrastructure support. Hospital structure fixed costs, such as energy, equipment maintenance, third party and service contracts, software licenses, taxes and general materials were estimated per ward, based on the total amount of patients taking in charge by the ward in the investigating period.

The analysis did not consider medications’ compassionate use and all donations in terms of PPE and equipment, with any economic impact for hospitals. On the contrary, PPE directly acquired by hospitals or by means of the Civil Protection, have been accordingly assessed.

All the above items of healthcare expenditure derived from the anonymous administrative and accounting flows by cost center- provided by the management control of the hospitals involved, thus estimating the COVID-19 resources absorption.

Once having collected the anonymous flows, the average per day costs and the average most frequent clinical pathways (considering the internal transfers between wards, based on the patient’s clinical improvement or deterioration), were accordingly valorized concerning the specific clinical condition of the COVID-19 positive patients requiring a different hospitalization.

1. Low-complexity medical hospitalization, considering a hospital stay without the need of any non-invasive ventilation.
2. Medium-complexity hospitalization, with the presence of hospital beds equipped with C-PAP or non-invasive ventilation.
3. High-complexity intensive care unit hospitalization, for treating COVID-19 patients, requiring invasive ventilation, through intubation.

The total direct medical cost per patient was calculated, based on the level of care and the related average length of stay.

In conclusions, Bayesian statistics were performed [25, 26]. Gamma distributions were accordingly developed, to verify the robustness of the results in presence of uncertainty factors, concerning the cost related to the single hospital stay per day, the entire clinical pathway of the COVID-19 patients, and the overall length of stay, considering the different level of care and patient’s complexity.

## Results

The economic evaluation of COVID-19 positive patients’ clinical pathways was assessed within 6 Italian public Hospitals managing COVID-19 patients, involving 34 wards devoted to the care and the treatment of infected individuals, requiring a hospitalization.

Among them, most wards (50%) were devoted to a medium-complexity hospitalization, followed by 35.29% characterized by a low-complexity hospitalization. 14.71% were intensive care units (ICUs).

COVID-19 patients requiring a hospitalization were on average 695 for any hospitals involved (4,170 patients in total), most of them being males (76%) and with an average age of 62.95 years (ranging from a minimum of 36 years to a maximum of 95 years). During the year 2020, 49.89%, 40.14% and 9.96% of patients requested a medium-complexity (average length of stay equal to 11.39 days), a low-complexity (average length of stay equal to 12.16 days) and a high-complexity (average length of stay 11.31 days) hospitalization, respectively.

From an economic perspective, Table 1 reported the economic evaluation of a single hospitalization day, stratified by level of care and patient’s complexity. Out of laboratory exams and diagnostic procedures costs, 586.43€ are related to the COVID-19 positivity confirmation phase at hospital level, in the Emergency Department. On the other hand, the higher the complexity of care, the higher the hospitalization cost per day (low-complexity = 475.86€; medium-complexity = 700.20€; high-complexity = 1,401.65€).

**Table 1:** The cost of a single hospitalization day

Items of healthcare expenditure	Low-complexity hospitalization	Medium-complexity hospitalization	High-complexity hospitalization
Laboratory Exams	433.71 €	526.92 €	1,120.95 €
Diagnostic procedures	699.99 €	853.59 €	2,129.33 €
Human resources	2,159.54 €	3,189.74 €	5,645.59 €
Oxygen Therapy	1,006.24 €	1,362.37 €	2,283.52 €
Drugs	176.79 €	322.33 €	1,407.96 €
PPE	149.78 €	157.71 €	264.98 €
Equipment	0.54 €	1.52 €	2.83 €
Meal and Cleaning services	195.28 €	230.42 €	360.08 €
General and fixed costs	964.37 €	1,328.92 €	2.643,05 €
**Total cost related to hospitalisation**	**5,786.25 €**	**7,973.52 €**	**15.858,29 €**
**Cost related to the single hospitalization day**	**475.86 €**	**700.20 €**	**1,401.65 €**

The most impacting items of costs are related to medical and nursing assistance (> 40%) and diagnostic procedures conducted at hospital beds (> 17%), independently from the level of care analyzed. Focusing on the assistance time per patient, the analysis conducted revealed that the average time spent by a clinician (in terms of average minutes per day per patient) is equal to 147 min, 180 min, and 240 min, within a low-complexity hospitalization, medium-complexity hospitalization, and a high-complexity hospitalization, respectively. The same increasing trend could be observed for both nurses and assistant healthcare professionals involved in the care of COVID-19 patients. Considering a low-complexity hospitalization, nurses spent on average 120 min per day per patient and assistant healthcare professionals spent on average 50 min per day per patient. Within a medium-complexity area, nurses spent on average 300 min per day per patient and assistant healthcare professionals spent on average 90 min per day per patient. Instead, patient hospitalized within an ICU required an assistance equal to 600 min per day provided by a nurse and equal to 120 min per day provided by an assistant healthcare professional.

On the other hand, the impact of the new equipment acquisition cost, was marginal within the three settings investigated (0.02%). In general, the hospitals involved supported further investments for the acquisition of both PPE (133,982€ on average) and ventilation equipment (561,688€ on average).

**Fig. 1:**
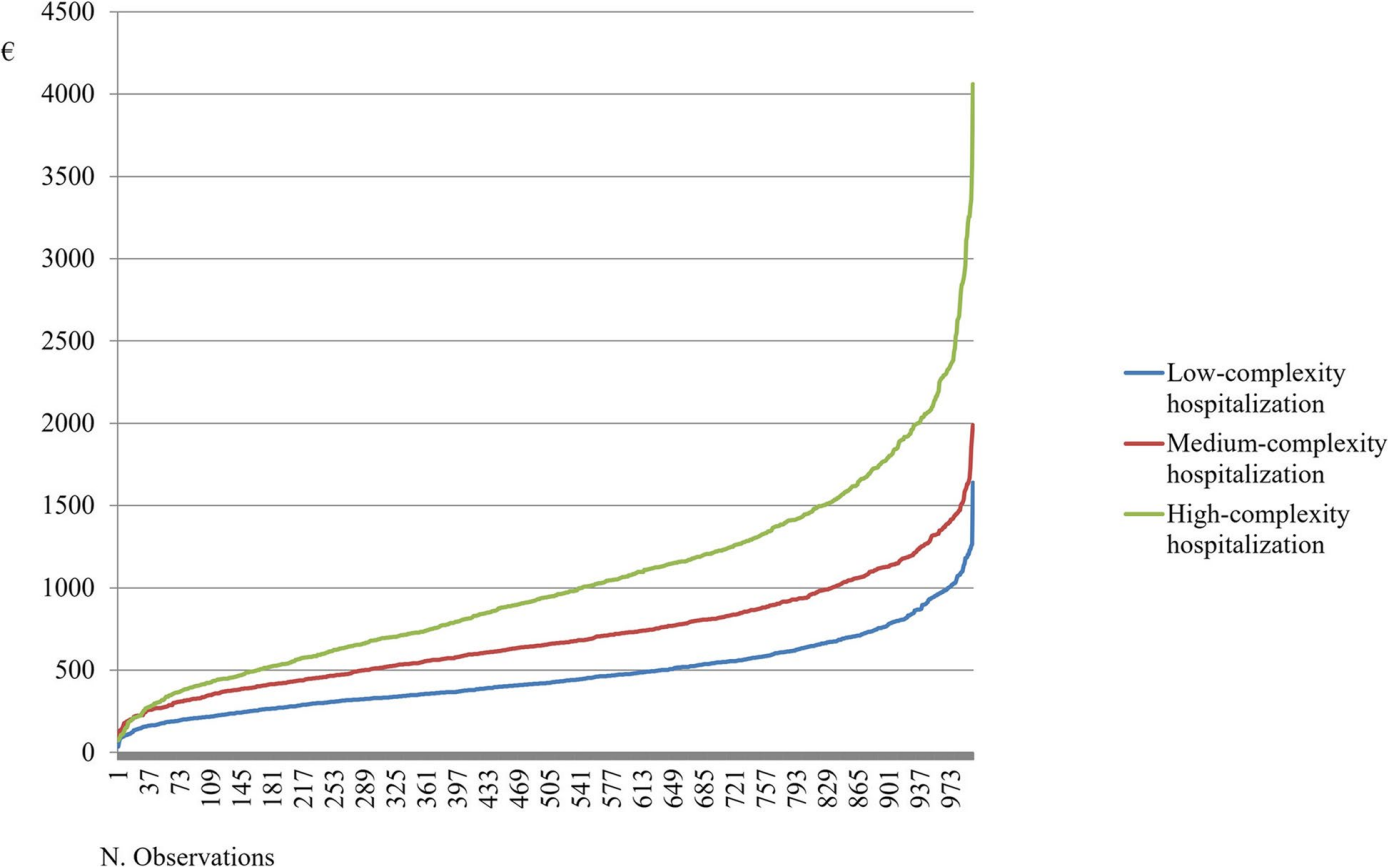
Gamma distributions for the cost of single hospitalization day

Gamma distributions related to the single hospitalization day costs (Fig. 1) reported that a high-complexity hospitalization required higher economic resources than a medium and a low-complexity hospitalization, respectively in 97% of cases and 100% of cases. On the contrary, a single stay in a medium-complexity area presented a probability equal to 3% to absorb higher economic resources than a single hospital day spent within a high-complexity area.

**Fig. 2:**
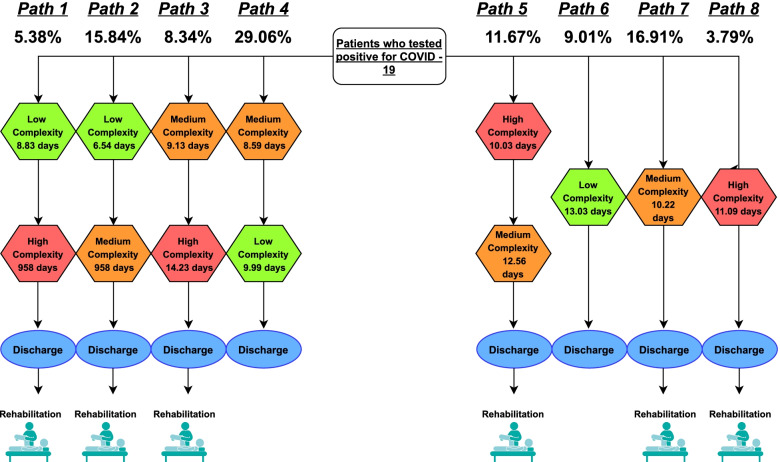
Graphic representation of the most prevalent clinical pathway

If the entire COVID-19 positive patients’ clinical pathway was analyzed, considering the potential internal ward transfer, due to an improvement or a deterioration of the patients’ clinical conditions, 8 main clinical pathways emerged (Fig. 2).

Table 2 reports the economic evaluation of the main clinical pathways performed by a COVID-19 positive patient within the hospital setting. While the first three pathways refer to a worsening of the patient's clinical condition, both pathway 4 and pathway 5 refers, instead, to an improvement in the patient's condition.

**Table 2:** Economic evaluation of the main COVID-19 clinical pathways

**Clinical Pathway**	**Complexity Area of Access**	**Length of stay in the area of Access** **(# days)**	**Internal Ward transfer**	**Length of stay in the Internal Ward transfer** **(# days)**	**Economic evaluation of the clinical pathway** **(€)**	**Total Length of stay** **(# days)**
Clinical Pathway #1 (5.38% of patients)	Low-complexity hospitalization	8.8	High-complexity hospitalization	9.6	**17,629.63€**	**18.4**
Clinical Pathway #2 (15.84% of patients)	Low-complexity hospitalization	6.5	Medium-complexity hospitalization	11.2	**10,941.41€**	**17.7**
Clinical Pathway #3 (8.34% of patients)	Medium-complexity hospitalization	9.1	High-complexity hospitalization	14.2	**26,339.20€**	**23.4**
Clinical Pathway #4 (29.06% of patients)	Medium-complexity hospitalization	8.6	Low-complexity hospitalization	10.0	**10,778.04€**	**18.6**
Clinical Pathway #5 (11.67% of patients)	High-complexity hospitalization	10.0	Medium-complexity hospitalization	12.6	**22,857.28€**	**22.6**
Pathway #6 (9.01% of patients)	Low-complexity hospitalization				**6,198.02€**	13.0
Pathway #7 (16.91% of patients)	Medium-complexity hospitalization				**7,152.53€**	10.2
Pathway #8 (3.79% of patients)	High-complexity hospitalization				**15,544.32€**	11.0

Furthermore, 29.71% of the patients presents an overall hospital stay in the same clinical ward, without any changes in their clinical condition.

**Fig. 3:**
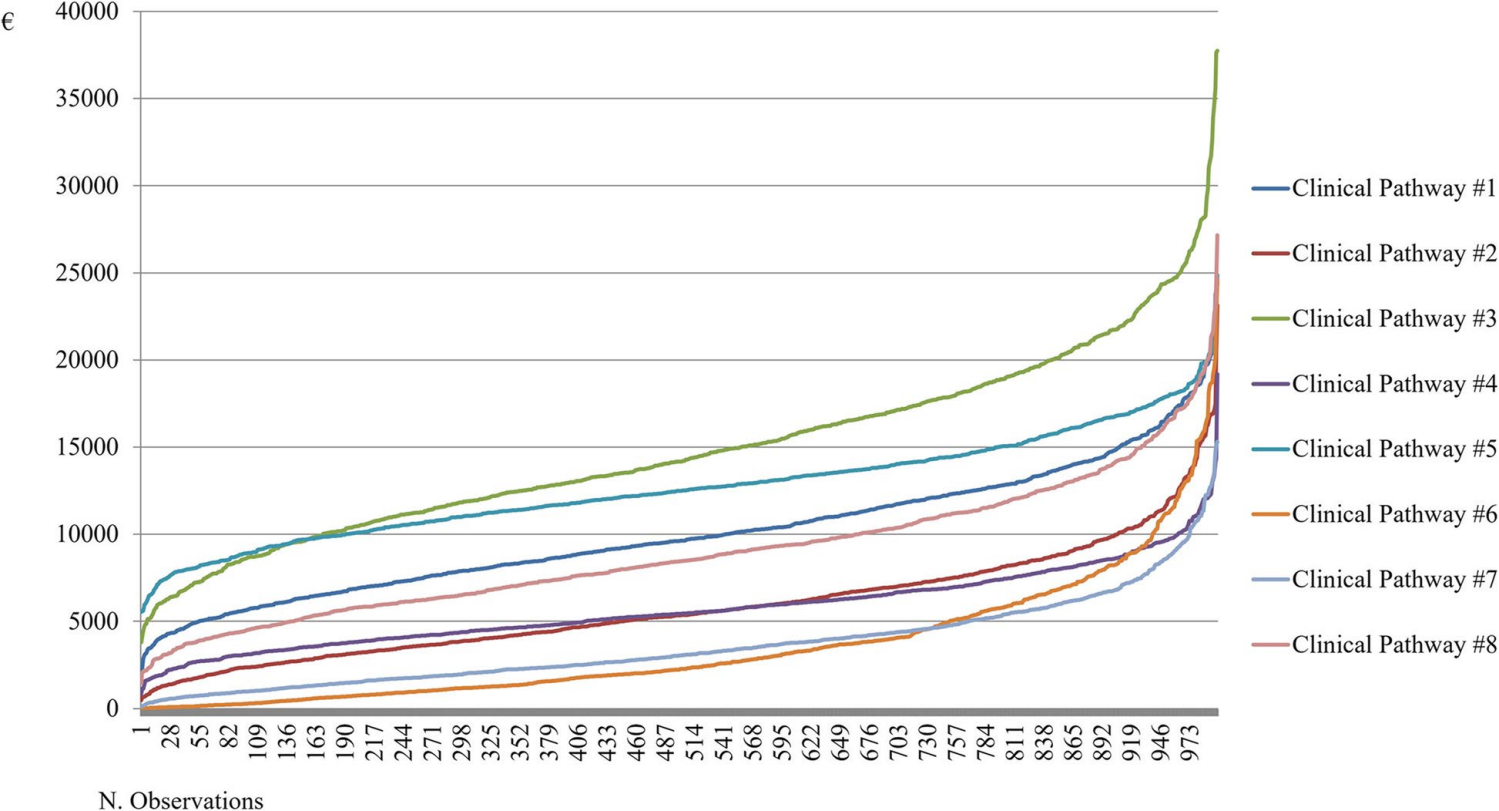
Gamma distributions for the cost of the entire clinical pathway of a COVID-19 positive patient

**Fig. 4:**
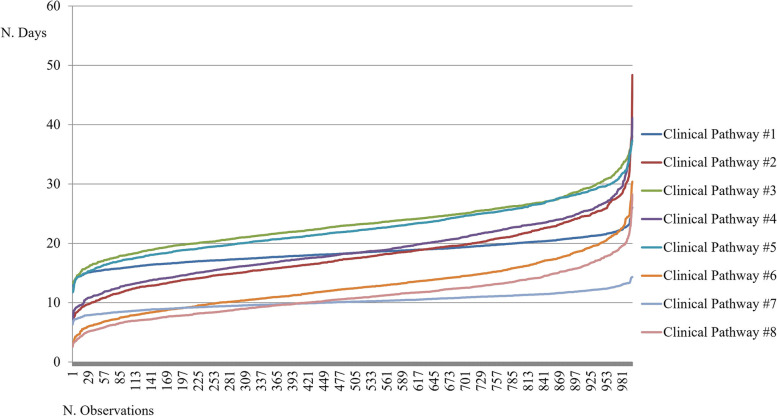
Gamma distributions for the overall length of stay of a COVID-19 positive patient

According to the above, Figs. 3 and 4 showed Gamma distributions related to the entire clinical pathway hospital costs and the related length of stay.

Clinical Pathway #6 and #7 presented a lower hospitalization cost, with a probability for patients hospitalized in only a low-complexity area equal to 79.8% to absorb fewer economic resources than those hospitalized in only a medium-complexity area.

Both Clinical Pathway #3 and Clinical Pathway #5 always required higher economic resources than the others. As for the economic assessment of the single hospitalization day, patients being hospitalized only in a low-complexity area presented a probability of 26.4% to absorb more economic resources than patients requiring a medium-complexity hospitalization, for their proper clinical management.

In the comparison between Clinical Pathway #2 and #4, patients experiencing a clinical improvement (thus being transferred from a medium-complexity to a low-complexity area) presented a probability of 43.7% to spend fewer resources absorption than patients with a deterioration of their clinical conditions (thus being transferred from a low-complexity to a medium-complexity area). The same trend emerged in the comparison between Clinical Pathway #3 and #5, although patients have passed through the same wards, characterized by the same intensity / complexity of care, the fact of having a clinical improvement of the patient has resulted in a reduction of economic resources equal to € 3,481.93, with an overall probability to absorb lower resources equal to 84.5%.

All the proposed considerations related to the clinical pathways’ costs, could be replicated for the analysis of the length of stay item, due to the collinearity of these two variables, as presented in Fig. 4.

An additional analysis was conducted with the inclusion of the rehabilitation pathway. Even in COVID-19 positive patients, as in other major respiratory diseases, cardio-respiratory rehabilitation plays an important role in promoting the resumption of daily life and in improving reintegration into the community, by increasing mobility, autonomy, and the patient health-related quality of life.

In this regard, for the definition of the rehabilitation process, both the in-patients (with a rehabilitative hospitalization) and the outpatients (without a rehabilitative hospitalization, but with a prescription of at least three months of activities) procedures were considered.

Based on these arguments, the cost related to the rehabilitation process performed as an in-patient activity is equal to 5,802€, to be attributed to hospital clinical pathways, presenting patients who are discharged from a high intensity/complexity of care (17.51% of the total). On the other hand, a cost equal to 2,960.60€ was evaluated for patients who are discharged from a medium intensity/complexity of care, for whom an outpatient rehabilitation activity is required (44.42%). A percentage equal to 38.07% of patients, did not experience any rehabilitation pathway, due to a discharge from low intensity/complexity of care.

In this view, it emerged that rehabilitation presents an impact ranging from 11 to 29% of the total costs sustained for the proper management of COVID-19 patients (Table 3).

**Table 3:** Economic evaluation of the main COVID-19 clinical pathway, with the inclusion of Rehabilitation costs

**Clinical Pathway**	**Economic evaluation of the hospital clinical pathway [€]**	**Rehabilitation clinical pathway** **[€]**	**Total costs (Hospital pathway + Rehabilitation clinical pathway)** **[€]**	**Rehabilitation pathway impact on total costs** **[%]**
Clinical Pathway #1 (5.38% of patients)	17,629.63€	5,802.00€	23,431.63€	24.76%
Clinical Pathway #2 (15.84% of patients)	10,941.41€	2,960.60€	13,902.01€	21.30%
Clinical Pathway #3 (8.34% of patients)	26,339.20€	5,802.00€	32,141.20€	18.05%
Clinical Pathway #4 (29.06% of patients)	10,778.04€	0.00€	10,778.04€	0.00%
Clinical Pathway #5 (11.67% of patients)	22,857.28€	2,960.60€	25,817.88€	11.47%
Pathway #6 (9.01% of patients)	3,421.47€	0.00€	6,198.02€	0.00%
Pathway #7 (16.91% of patients)	3,717.33€	2,960.60€	10,113.13€	29.27%
Pathway #8 (3.79% of patients)	8,900.42€	5,802.00€	21,346.32€	27.18%

## Discussion

Italy has been the first-hit European country to face the outbreak of COVID-19, with a consequent intensification of demand on the healthcare system, resulting in a critical shortage of hospital resources (hospital beds, ICU beds, ventilators, healthcare professional workforce).

To the best of the authors’ knowledge, the present paper could represent the first attempt to investigate the economic resources absorption devoted to the hospital management of COVID-19 positive patients, thus defining the costs directly sustained by hospitals, based on real-word data within the specific Italian setting.

In the presentation of a comprehensive picture, with regard to the economic evaluation of COVID-19 pandemic in six hospitals in Italy, the cost data highlighted differences in resources utilization between patients presenting moderate-to-severe symptoms, versus critical cases who required ICU admission, thus providing relevant information that could represent the baseline cost for COVID-19 hospital management, independently from the concomitant diseases developed by a patient.

The attention should be focused on the cost related to a single hospitalization day within the three different settings, reporting that the higher the intensity of care, the higher the cost, ranging from 475.86€ considering a low-complexity hospitalization to 1,401.65€ considering an ICU hospitalization. A day spent in a medium-complexity hospitalization absorbed 700.20€. The above information, rely on real-word evidence directly derived from administrative and accounting flows, could be replicable for any hospitals taking in charge COVID-19 positive patients.

The results here presented could be consistent with few literatures evidence produced on the topic: for example, in Greece, the hospital per day cost in general ward is estimated equal to 443.1€, while cost per day in ICU at 2,245.5€ [27], whereas in the US the cost of treating COVID-19 pneumonia with complications or comorbidity presents an economic amount of 13,767$ [28]. Similar results to those presented, were found in other Countries, concerning the average length of stay. In Turkey, for example, a retrospective analysis conducted from the first peak of the pandemic reported a mean length of stay equal to 9.1 days, ranging from 8.0 days for patients hospitalized in medicine wards, to 14.8 days for patients hospitalized in the ICU [29].

In general terms, the few available evidence on the economic resources’ absorption, concerning COVID-19 clinical pathway, are poor and limited, due to the existence of differences in methods, technologies, healthcare costs and nature of services delivery. Nevertheless, all this evidence declared that the burden of COVID-19 on the healthcare systems in terms of resources’ use and costs, was substantial [30–32].

Assuming a critical point of view, the current research activity present three main limitations. At first, in the economic evaluation of the COVID-19, many variables such as comorbidities (e.g., diabetes mellitus, asthma, cancer, hypertension, chronic kidney disease), smoking status, and occupation were not investigated, which may have changed our findings, if they had been added and evaluated in our analyses. Additionally, the study did not consider the changes in the treatment protocols and their potential impact on the length of stay, thus comparing hospital costs based on the treatment administered to COVID-19 patients. Third, this study was conducted from the perspective of hospitals, and did not take into consideration other important costs, such as productivity losses, “lockdown” costs, as well as hospital revenue shortfalls due to the cancelation of elective procedures or because patients avoided being admitted to hospitals [33]. For example, in the United Kingdom, schools and business closures resulted together in a total burden equal to 668 billion£ (29.2% of GDP) on the British economy [34].

According to the above, further research could be conducted to better understand any possible correlation with COVID-19 and complications occurred, as well as any positive impact of the novel medications on the improvement of the clinical condition of the COVID-19 positive patients, with important benefits on the length of stay, and potential reduction in the overall direct healthcare costs [7]. Economic data collected for the conduction of the present study could be integrated with COVID-19 patients’ complete demographic and clinical information, during a retrospective observation study: in this view, multiple linear regression models would be performed to estimate the relationships between the hospitalization costs, and the potential patients’ factors.

In conclusions, the information obtained could represent not only the baseline cost for COVID-19 positive patients hospital management, but also could support hospitals’ benchmarking activities. A detailed segmentation of all costs and pathways could help policymakers in addressing regulations, to make a more efficient and effective use of scarce resources. In the Italian setting, no consensus exists regarding a standardized reimbursement tariff for such disease; the most used DRGs (Diagnosis related Group) for coding the COVID-19 hospitalization is DRG 079 (respiratory infections), which presents a national cost equal to 5,744€. Based on the Decree 12/08/2021 an increase of such tariff equal to 3,713€ would be applied if no ICU hospitalization occurred, whereas an increase of 9,617€ would be observed if the patient experienced at least one day in an ICU bed. Based on these considerations and in the comparison between the reimbursement tariffs available nowadays and the costs directly sustained by hospitals for the proper care of hospitalized COVID-19 patients, it emerged that the reimbursement tariffs are not able to cover all the hospital management costs, in most of the clinical pathways here presented. Hospital costs present a greater economic value ranging from a minimum of 12.26% (Clinical Pathway #5) to a maximum of 41.68% (Clinical Pathway #3). The proposed reimbursement tariffs could be economically sustainable only if the COVID-19 patient will experience a hospitalization in a low-complexity area, or in a medium-complexity area, without any internal ward transfers.

Based on the above, these economic results could be useful in the definition of the proper reimbursement tariffs devoted to the hospitalization of COVID-19 positive patients, based on their clinical conditions, thus being more appropriate and sustainable for all the countries based on a National Healthcare System.

## Data Availability

The datasets used and/or analysed during the current study are available from the corresponding author on reasonable request.

## Abbreviations

COVID-19: Coronavirus disease-2019
DRG: Diagnosis Related Group
ICD: International Classification of Diseases
ICU(s): Intensive Care Unit(s)
PPE: Personal Protective equipment
SARS-CoV-2: Severe acute respiratory syndrome coronavirus-2
TDABC: Time-driven activity-based costing approach
